# A new Seasonal Difference Space-Time Autoregressive Integrated Moving Average (SD-STARIMA) model and spatiotemporal trend prediction analysis for Hemorrhagic Fever with Renal Syndrome (HFRS)

**DOI:** 10.1371/journal.pone.0207518

**Published:** 2018-11-26

**Authors:** Youlin Zhao, Liang Ge, Yijun Zhou, Zhongfang Sun, Erlong Zheng, Xingmeng Wang, Yongchun Huang, Huiping Cheng

**Affiliations:** 1 Business School of Hohai University, Nanjing city, Jiangsu Province, PR China; 2 Tianjin Institute of Surveying and Mapping, Tianjin city, PR China; 3 School of Economics and Management, Hubei University of Technology, Wuhan,Hubei Province, PR China; University of Waikato, NEW ZEALAND

## Abstract

Hemorrhagic fever with renal syndrome (HFRS) is a naturally-occurring, fecally transmitted disease caused by a Hantavirus (HV). It is extremely damaging to human health and results in many deaths annually, especially in Hubei Province, China. One of the primary characteristics of HFRS is the spatiotemporal heterogeneity of its occurrence, with notable seasonal differences. In view of this heterogeneity, the present study suggests that there is a need to focus on trend simulation and the spatiotemporal prediction of HFRS outbreaks. To facilitate this, we constructed a new Seasonal Difference Space-Time Autoregressive Integrated Moving Average (SD-STARIMA) model. The SD-STARIMA model is based on the spatial and temporal characteristics of the Space-Time Autoregressive Integrated Moving Average (STARMA) model first developed by Cliff and Ord in 1974, which has proven useful in modelling the temporal aspects of spatially located data. This model can simulate the trends in HFRS epidemics, taking into consideration both spatial and temporal variations. The SD-STARIMA model is also able to make seasonal difference calculations to eliminate temporally non-stationary problems that are present in the HFRS data. Experiments have demonstrated that the proposed SD-STARIMA model offers notably better prediction accuracy, especially for spatiotemporal series data with seasonal distribution characteristics.

## Introduction

Hemorrhagic Fever with Renal Syndrome (HFRS) is a serious infectious disease that is mainly caused by a Hantavirus (HTNV) and the Seoul virus (SEOV) [1–4]. The clinical symptoms for HFRS are fever, hemorrhaging and renal dysfunction and it can result in long-term kidney damage, hypotension and even death. HFRS has s distribution across a number of countries. China is the most seriously affected, accounting for more than 90% of the world's cases of HFRS [5–9]. Within China, however, one province in particular, Hubei, has become the most seriously affected area of all in recent years. Since the first case of HFRS was reported in Hubei in 1957, HFRS epidemics have expanded and reached a high point in 1983 with 23,943 cases. From 1980 to 2009, the number of HFRS cases in Hubei Province totaled 104,467. The spread of HFRS has had a significant impact on social stability and human health [10–12].

Spatial and temporal statistical methods have been used to discover the spatial and temporal distribution and clustering characteristics of HFRS across a number of different locations [13], including Buenos Aires in Argentina [14], Germany [15] and Brussels in Belgium [16]. In China, a Kulldorff spatial scan statistic has been used to try and identify the clustering of HFRS, drawing upon data spanning the period 1980 to 2009 [17]. A Gaussian GWR model has also been used to try and identify the factors influencing HFRS transmission (such as meteorological factors, rodent density, surface mean elevation, water area and human population density) drawing upon data from Hubei that was collected between 2011 and 2015 [18]. Moran’s *I* index was adopted for a global spatial autocorrelation analysis that sought to identify the overall spatiotemporal pattern of HFRS outbreaks in Hubei between 2005 and 2014, and Spearman's rank correlation analysis was used at the same time to explore the possible factors influencing the epidemics, such as the weather and the area’s geography [19]. Cross-correlation analysis has also been used to assess a possible association with meteorological variables and a time-series Poisson regression model was adopted to examine the independent contribution of meteorological variables to HFRS transmission in both Elunchun and Molidawahaner counties in Northeastern China between 1997 and 2007 [20]. Alongside of this, a generalized additive model with penalized smoothing splines has been used to examine the effect of meteorological factors on the occurrence of HFRS in Jiaonan between 2006 and 2011 [21].

Identifying the spatial and temporal distribution of HFRS can help with analyzing and evaluating the trends in HFRS outbreaks, thus leading to the adoption of more effective measures for the prevention and control of the disease. HFRS, however, has a frustrating degree of spatiotemporal heterogeneity and seasonal variation [19]. So, in order to conduct a better analysis of HFRS distribution and to acquire a more accurate means of prediction, the construction of a space-time model seems to be called for. Space-time modeling refers to the process of finding an analytical method to model and predict the value of an unrecorded space-time position based on given spatiotemporal data [22]. Space-time modeling is a spatial expansion of time series modelling and the factors influencing the attribute values of unobserved space-time positions bring together the spatial and temporal factors associated with single time series modeling, single spatial modeling and spatiotemporal modeling.

The most representative single time series model is Autoregressive Integrated Moving Averages (ARIMA). This analyzes the time series of historical data and obtains the model with the optimal fit for predicting events that will occur in the short term [23] [24]. An ARIMA model shows time series data that is related to both sequentially lagged variables and their errors. ARIMA models have been used several times for the prediction of HFRS outbreaks [25,23,26–28], which indicates that this model is a good fit here as well for the forecasting of outbreaks.

For the single spatial modeling, there are space autoregressive models and space moving average models. Based on a spatial weight matrix, these models study the quantization measure of neighboring spatial units [29].

Drawing upon time and space series modeling, the geographer A.D. Cliff and the statistician J.K. Ord, originally proposed in 1974 a space-time series modeling framework [22] that is essentially a spatial expansion of the time series model. It combines Spatial Autocorrelation (SAR), a Spatial Moving Average (SMA) and Spatial Regression (SR). A large number of studies had shown that, whilst the ARIMA model provided better fitting results for data with a relatively stable temporal distribution and no strong spatial autocorrelation, its effectiveness for prediction relating to spatiotemporally heterogenous sample data was much weaker [30–32]. Cliff and Ord’s Spatiotemporal Autoregressive Integrated Moving Average (STARIMA) extended beyond the ARIMA model [33]. The STARIMA model provides a space-time autocorrelation function (ST-ACF) and a space-time partial correlation function (ST-PACF) to address the problem of measuring spatiotemporal correlations. It also introduced a spatiotemporal lag operator that makes it capable of simultaneously extrapolating and predicting multiple spatial units [34]. The STARIMA model was subsequently proved to offer high estimation performance when applied to a case study of the regional deposits of commercial banks operating in Turkey using non-linear estimators [35]. The STARIMA model has also been applied to rainfall and waterlogging process simulation and to short-term forecasting. Here, it offers improved prediction accuracy and reliability when compared to traditional hydro model simulation and prediction [36]. Outside of this, STARIMA models have been applied to traffic prediction, environment variable prediction and in social and economic analyses [37–41].

Research has indicated that HFRS has a characteristic seasonal or cyclic time series-based occurrence [42,11]. In our previous work, a Seasonal Difference—Geographically and Temporally Weighted Regression (SD-GTWR) model was developed as an extension of the GTWR model that sought to use seasonal difference to get stabilized data [43]. Seasonal difference was used to deal with a non-stationary time series with seasonal distribution characteristics. Following on from this research, we constructed a Seasonal Difference—Space-Time Auto Regressive Integrated Moving Average (SD-STARIMA) model that is based on STARIMA. Time serials analysis and autocorrelation analysis were conducted to ensure the feasibility of using a seasonal difference approach. The STARIMA model is a prerequisite for advanced seasonal difference modeling and analysis. In our previous research, we found that from 1980 to 2000 [17] and from 2005 to 2014 [19] the HFRS cases in Hubei Province displayed a bimodal seasonal distribution pattern rather than a linear distribution. Seasonal difference calculations for HFRS incidence in Hubei using SD-STARIMA offer the prospect of improving the accuracy of previous space-time series models. The main contribution of this paper is the development of a new SD-STARIMA model that is able to bring seasonal difference calculations to bear in a way that will eliminate the non-stationary temporality problem found in HFRS data. Estimation results from the SD-STARIMA model show it to be more accurate than other models such as ARIMA and STARIMA. This confirms its potential to contribute to the prevention and control of HFRS.

## Study data and analysis

### Study data

The area focused on in this study is Hubei Province in central-southern China. The data covers the period from 2005 to 2014. In the past 30 years, the data during this decade is the most representative and 2014 is the most recent year for which detailed data is available. Basic geographic data about Hubei Province was collected from the Chinese National Administrator of Surveying, Mapping and Geo-Information. HFRS case data was provided by the Hubei Province Center for Disease Control and Prevention and the Chinese Center for Disease Control and Prevention. The HFRS case data contains the monthly case values for each county. Meteorological data was obtained from the National Center for Environmental Prediction and the Hubei Meteorological Bureau. Human population density data was extracted from the Hubei Statistical Yearbook, which includes the annual population for each county.

### Seasonal characteristic analysis

**Fig 1:**
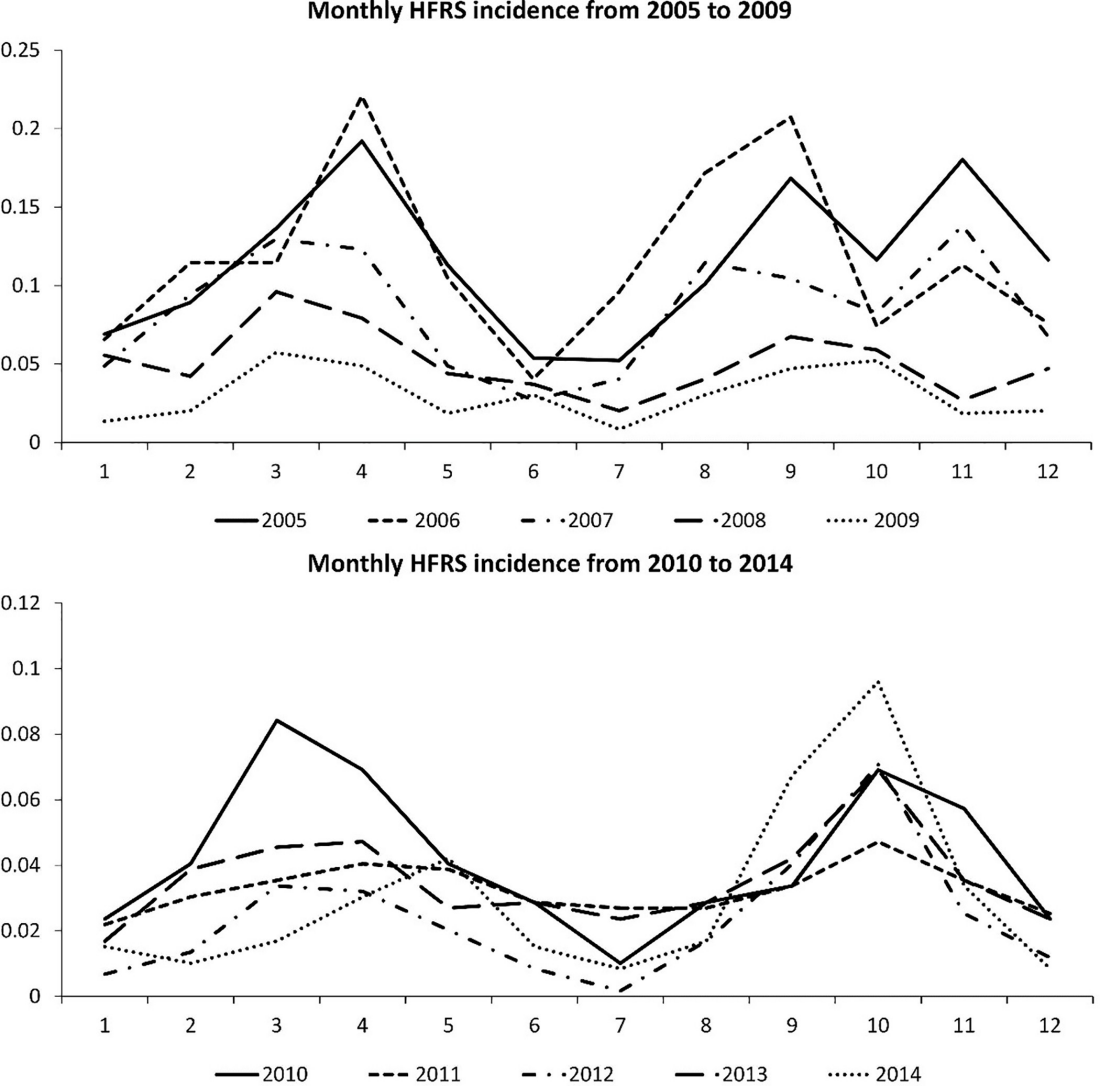
Monthly HFRS incidence from 2005 to 2014. (A) Average monthly HRFS incidence from 2005 to 2009. (B)Average monthly HRFS incidence from 2010 to 2014.

The monthly distribution pattern of HFRS in Hubei Province from 2005 to 2014 is shown in Fig 1. It can be seen that HFRS epidemics appear to have a bimodal distribution for each year (12 months), occurring around March and September. As a result, the time frame for the range of seasonal differences for each year has been narrowed down to 6 months for this study [44].

### Stationarity analysis of the HFRS incidence data

**Fig 2:**
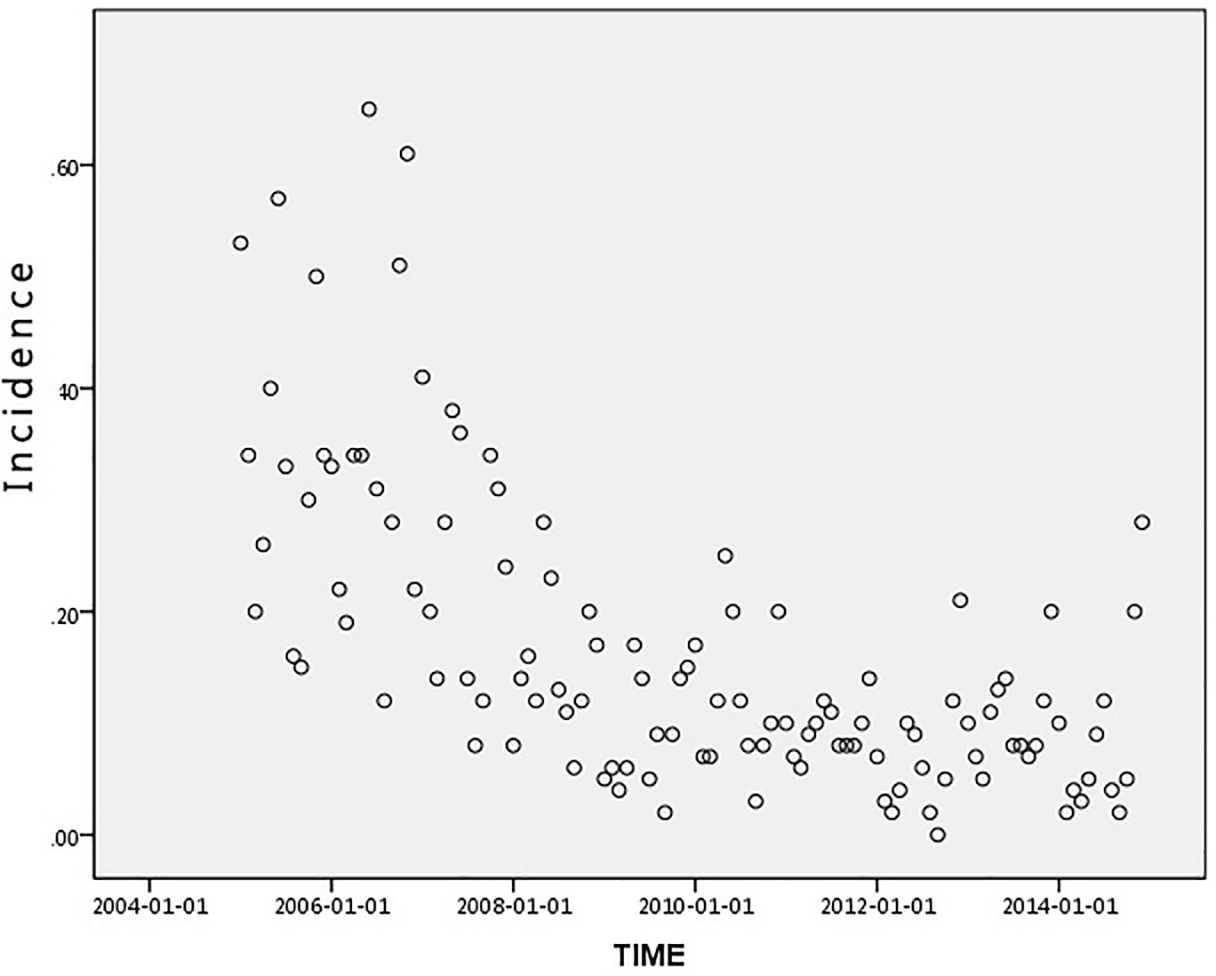
Scatter distribution of the HFRS incidence in Hubei Province from 2005 to 2014. Each plot shows the incidence of HFRS for a unique date.

**Fig 3:**
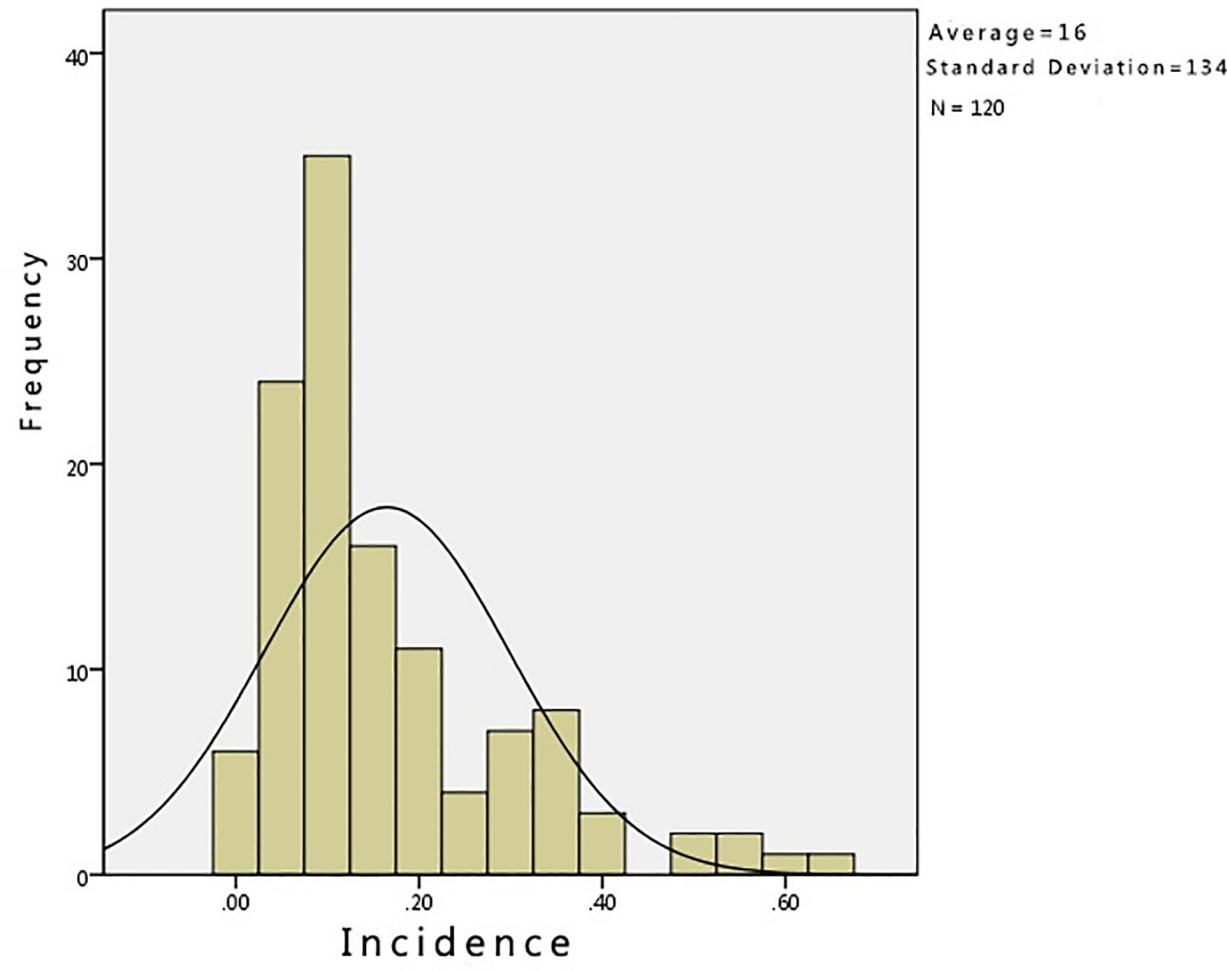
Normal distribution of the HFRS incidence data in Hubei Province from 2005 to 2014. Each column is an estimate of the probability distribution of the HFRS incidence.

**Fig 4:**
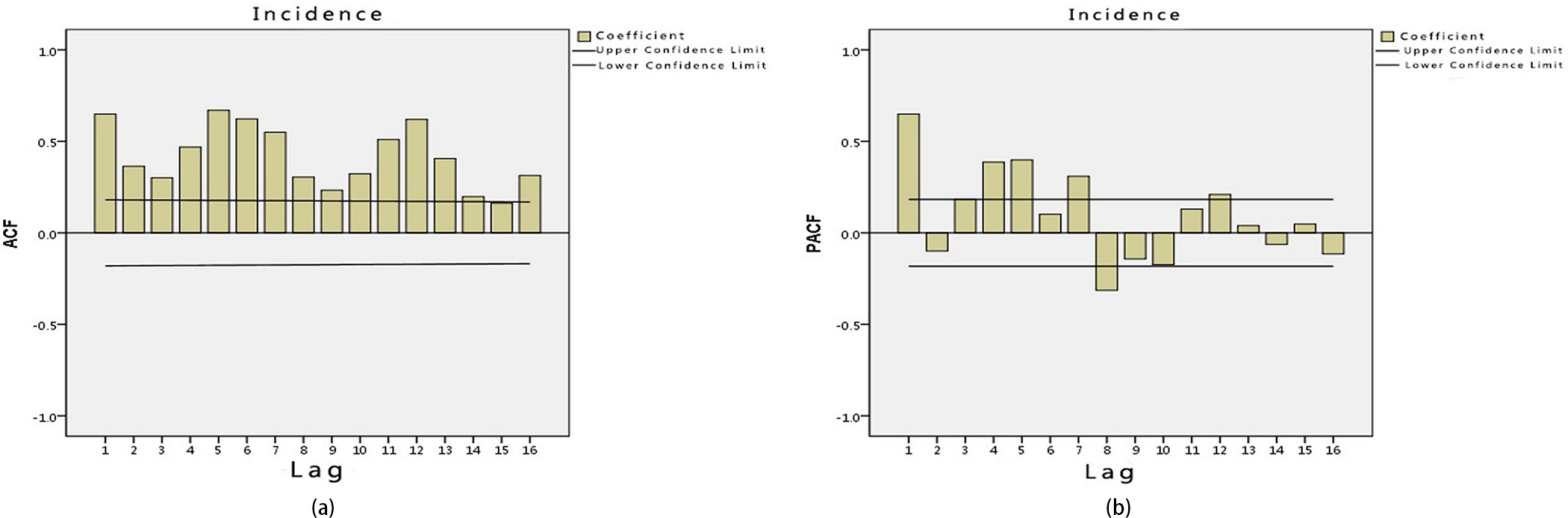
Correlation function values for the HFRS incidence data in Hubei Province from 2005 to 2014. **(a) Autocorrelation (b) Partial autocorrelation.** The ACF (autocorrelation function) values for HFRS incidence in each lag.

To arrive at a more effective time series analysis, it is necessary to identify the spatial and temporal series of the HFRS case data. Figs 2 and 3 show that the HFRS outbreak incidence in Hubei is clustered and does not meet the requirements of a normal distribution. In order to look for significant correlations in the HFRS outbreak distribution across the time series, an autocorrelation of the HFRS incidence time series data was undertaken using an autocorrelation graph. The autocorrelation graph and partial autocorrelation graph are plotted according to the autocorrelation and partial autocorrelation coefficients. In Fig 4(A), the abscissa is the number of lags and the ordinate is the ACF (autocorrelation function) value. The two lines in this figure represent the autocorrelation coefficient confidence interval of 95%. If there is no autocorrelation, the distribution pattern should be randomly distributed within the 95% confidence interval, without any fixed pattern and with the ACF values gradually tending to zero as the lag *k* increases. However, it can be seen from Fig 4(A) that the autocorrelation coefficient *r*_*k*_ does not do this. At the same time, it can be seen from Fig 4(B) that the partial correlation function value is larger at the 1^st^, 4^th^,5^th^,7^th^,8^th^ and 12^th^ order lag states. This indicates that there is periodicity in the time series. That being so, the time and space series for the HFRS case data in Hubei does not have a smooth time series.

Thus, according to the seasonal characteristics and stationarity analysis of the HFRS outbreaks presented above, the series for HFRS incidence distribution in Hubei Province is temporally unstable. As previously mentioned, a large number of studies have shown that ARIMA models are better able to fit data with a relatively stable time distribution and no strong spatial autocorrelation, but they are not so effective when there is spatiotemporal heterogeneity in the sample data [30–32]. This was the original reason for the development of Cliff and Ord’s, Spatiotemporal Autoregressive Integrated Moving Average (STARIMA) model [33]. However, the accuracy of this model is still limited for non-stationary series. In that case, there is a need for a new spatiotemporal series model that is capable of analyzing the seasonal characteristics and stationary distribution of the HFRS outbreaks in Hubei to improve the precision of the predictions.

## Construction of a seasonal difference Spatio-temporal autoregressive integrated moving average (SD-STARIMA) Model

By building upon both the ARIMA model and the STARIMA model, the SD-STARIMA model not only inherits the functions of STARIMA, but also has its own particular advantages. In this paper, the ARIMA analysis was conducted using SPSS 22 and the STARIMA analysis was conducted using R package. Construction and analysis of the SD-STARIMA model was conducted using MATLAB.

### Principles of the ARIMA model

ARIMA models are able to take into account changing trends, periodic changes, and random disturbances in a time series, so they are very useful for modeling a time series’ time dependence structure. In epidemiology, ARIMA models have been successfully applied to predict the incidence of a number of infectious diseases, such as influenza [45] and malaria [46], to mention but a few [47,48]. ARIMA (p,d,q) modeling of time series originated with the work of Box-Jenkins [24]. The model-building process was designed to take advantage of associations in the sequentially-lagged relationships that usually exist in periodically collected data [49]. The following were the parameters selected when fitting the ARIMA model: *p*, the order of autoregression; *d*, the integration parameter; and *q*, the order of the moving average. Autocorrelation function (ACF) and Partial autocorrelation function (PACF) graphs were used to identify the order of the moving average (MA) and the autoregressive (AR) terms included in the ARIMA model.

**Fig 5:**
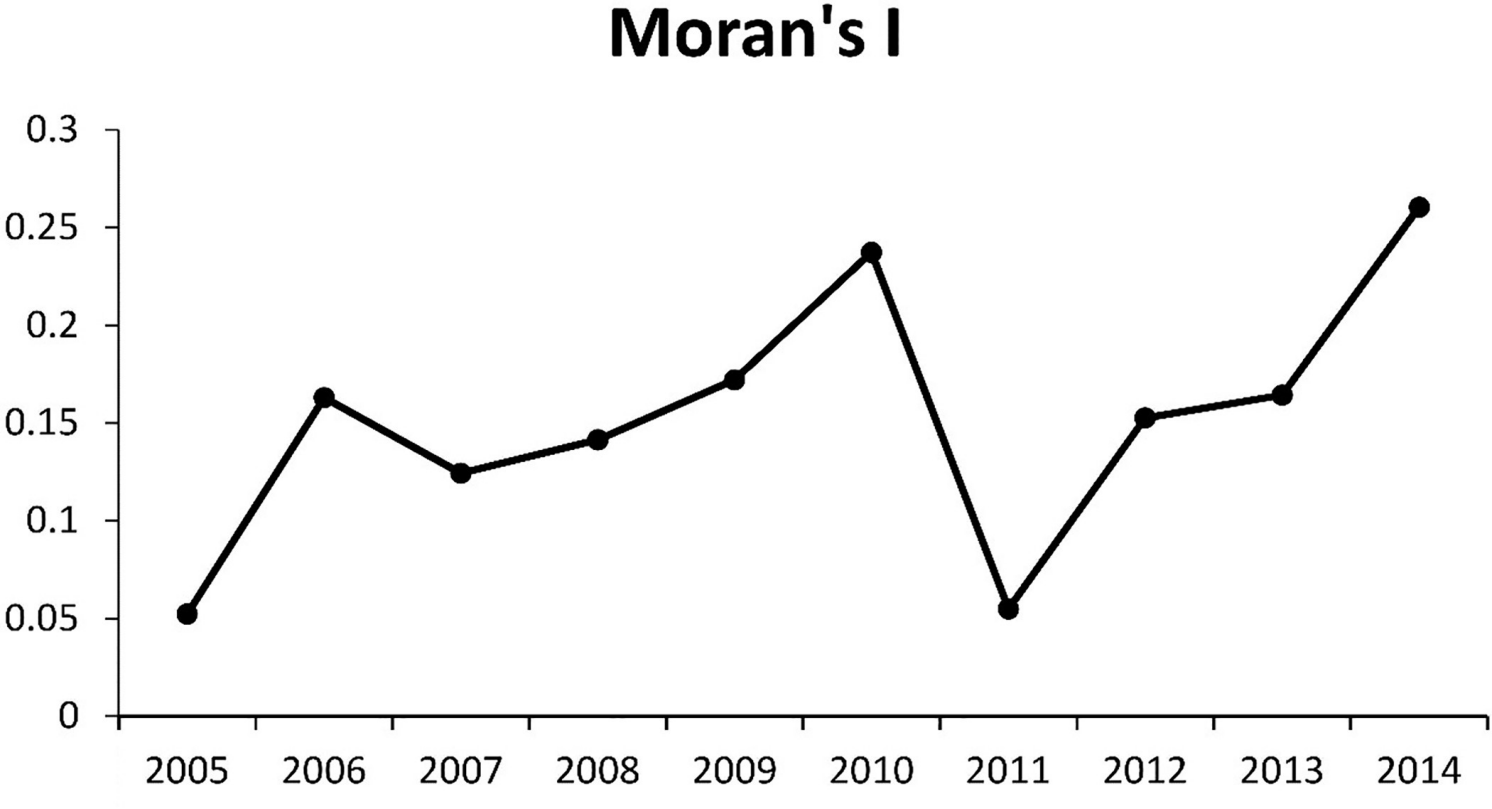
Distribution pattern of HFRS according to Moran's Index from 2005–2014. Each point represents the Moran’s *I* value for a specific year. All of the points are joined to indicate the trend of Moran’s *I* for the HFRS incidence in Hubei Province.

Fig 5 and Table 1 indicate the spatial autocorrelation results for Moran’s Index *I*. From this it can be concluded that the distribution of HFRS incidence in Hubei has spatial autocorrelation characteristics, so the trends for HFRS cannot be simulated using just time.

**Table 1:** Spatial autocorrelation results for the HFRS average annual incidence rate for each year in Hubei Province.

year	Moran's Index	Expected Index	Variance	z-score	p-value
2005	0.0523	-0.0133	0.0055	0.8844	0.3765
2006	0.1630	-0.0133	0.0050	2.4834	0.0130
2007	0.1242	0.1242	0.1242	2.2474	0.0246
2008	0.1413	-0.0133	0.0026	3.0412	0.0024
2009	0.1722	-0.0133	0.0045	2.7571	0.0058
2010	0.2374	-0.0133	0.0037	4.1336	0.0001
2011	0.0550	-0.0133	0.0030	1.2442	0.2134
2012	0.1526	-0.0133	0.0038	2.6845	0.0073
2013	0.1642	-0.0133	0.0027	3.4013	0.0007
2014	0.2604	-0.0133	0.0038	4.4143	0.0001

### Construction of the STARIMA model

The Space-time Autoregressive Integrated Moving Average model, STARIMA for short, is an extension of the ARIMA model. The STARIMA model class expresses: *z*_*i*_*(t)*; observations of the random variables at site *i*, *i = 1*,*2*,*…*, *N*; and time *t* as a weighted linear combination of past observations and errors, which may be lagged across both space and time. The basic mechanism for this representation is a hierarchical ordering of the neighbors of each site and a sequence of *N×N* weighting matrices, *W*^*(t)*^. Matrix *W*^*(t)*^ has elements *w*_*ij*_^*(t)*^ that are nonzero if and only if sites *i* and *j* are *l*^*th*^ order neighbors and *w*^*(o)*^ is defined to be a *N×N* identity matrix. Specifically, if you let *z(t)* be the *N×1* vector of observations at time *t*, the STARIMA model class can be expressed as follows [50]:

$$z\left(t\right)=-\sum_{k=1}^{p}\sum_{l=0}^{\lambda_{k}}\phi_{kl}W^{\left(l\right)}z\left(t-k\right)+\varepsilon \left(t\right)+\sum_{k=1}^{q}\sum_{l=0}^{m_{k}}\theta_{kl}W^{\left(l\right)}\varepsilon \left(t-k\right)$$ (1)

where *p* is the autoregressive order; *q* is the moving average order;*λ*_*k*_ is the spatial order of the *k*^*th*^ autoregressive term; *m*_*k*_ is the spatial order of the *k*^*th*^ moving average term;*Φ*_*kl*_ is the autoregressive parameter at temporal lag *k*and spatial lag *l*; *θ*_*kl*_ is the moving average parameter at temporal lag *k* and spatial lag *l*; *W*^*(t)*^ is the *N×N* matrix of weights for spatial order *l*; and *Ɛ(t)* is the random normally distributed error vector at time *t* [51].

E[ε(t)]=0

$$E\left[\varepsilon \left(t\right)\varepsilon \left(t+s\right)¢\right]=\{\begin{array}{c} G\;s=0\\ 0\;s\neq 0 \end{array}$$ (2)

E[ε(t)ε(t+s)′]=0fors>0.

This specific model is referred to as the STARIMA ($p_{\lambda_{1},\lambda_{2},\ldots ,\lambda_{p},}q_{m_{1},m_{2},\ldots m_{q}}$) model. Two special subclasses of the STARIMA model are of note. When *q = 0*, only autoregressive terms remain, in which case the model is called a space-time autoregressive or STAR model. Models that contain no autoregressive terms (*p = 0*) are referred to as STMA models.

### Construction of the SD-STARIMA model

By building upon the ARIMA model, the STARIMA model is able to evaluate the space functions pertaining to ARIMA. In essence, STARIMA is an extended linear regression model, so it can only describe linear autocorrelation results. That being so, STARIMA models are not well-suited to the prediction of the incidence of diseases with a seasonal epidemic pattern.

Our above analysis of the time series results for HFRS incidence in Hubei suggests that HFRS incidence does not have a stationary temporal distribution. ARIMA or ARIMA-based models need a stationary distribution of time series data as a prerequisite. In view of this, a seasonal difference method was used to eliminate the disruptive tendencies and get a stationary time series. The seasonal difference method amounts to being a way of getting a new time series by calculating the difference between various circles labeled L:

$$\Delta z_{t}=z_{t}-z_{t-1}=z_{t}-Lz_{t}=(1-L)z_{t}$$

$$\Delta^{2}z_{t}=\Delta z_{t}-\Delta z_{t-1}=(1-L)z_{t}-(1-L)z_{t-1}={\left(1-L\right)}^{2}z_{t}$$ (3)

$$\Delta^{d}z_{t}={\left(1-L\right)}^{d}z_{t}$$

A new series model can be obtained after the d-order difference calculation has finished. This can be formally defined as:

$$w_{t}=\Delta^{d}z_{t}={\left(1-L\right)}^{d}z_{t}$$ (4)

As previously mentioned, HFRS has specific spatially-distributed epidemics, with the seasonal epidemic pattern in Hubei being characteristically bimodal. The time frame for the seasonal difference calculation was set to 6 months. So, for the purposes of data stabilization by differential, the interval for each order of difference in the time series should be set to 6 months. The stationary series data used to establish the STARIMA model has three steps: identification; estimation; and diagnostic checking [52]. The novel SD-STARMA model proposed in this paper can be formally expressed as follows (9):

$$w(t)=-\sum_{k=1}^{p}\sum_{l=0}^{\lambda_{k}}\phi_{kl}W^{\left(l\right)}w(t-k)+\varepsilon (t)+\sum_{k=1}^{q}\sum_{l=0}^{m_{k}}\phi_{kl}W^{\left(l\right)}\varepsilon (t-k)$$ (5)

## Results and discussion

### Selection of the order of difference

The Augmented Dickey-Fuller test for unit root in level is conducted, the results are demonstrated in Table 2. It can be conducted in Table 2 that p value for ADF test is 0.07 which indicate that HFRS cases series is non-stationary distributed which *p*<0.07.

**Table 2:** Augmented Dickey-Fuller test statistic.

		t-Statistic	Prob.*
Augmented Dickey-Fuller test statistic		-2.721378	0.0734
Test critical values:	1% level	-3.485586	
	5% level	-2.885654	
	10% level	-2.579708	

* Lag Length: 11 (Automatic—based on SIC, maxlag = 12)

**Fig 6:**
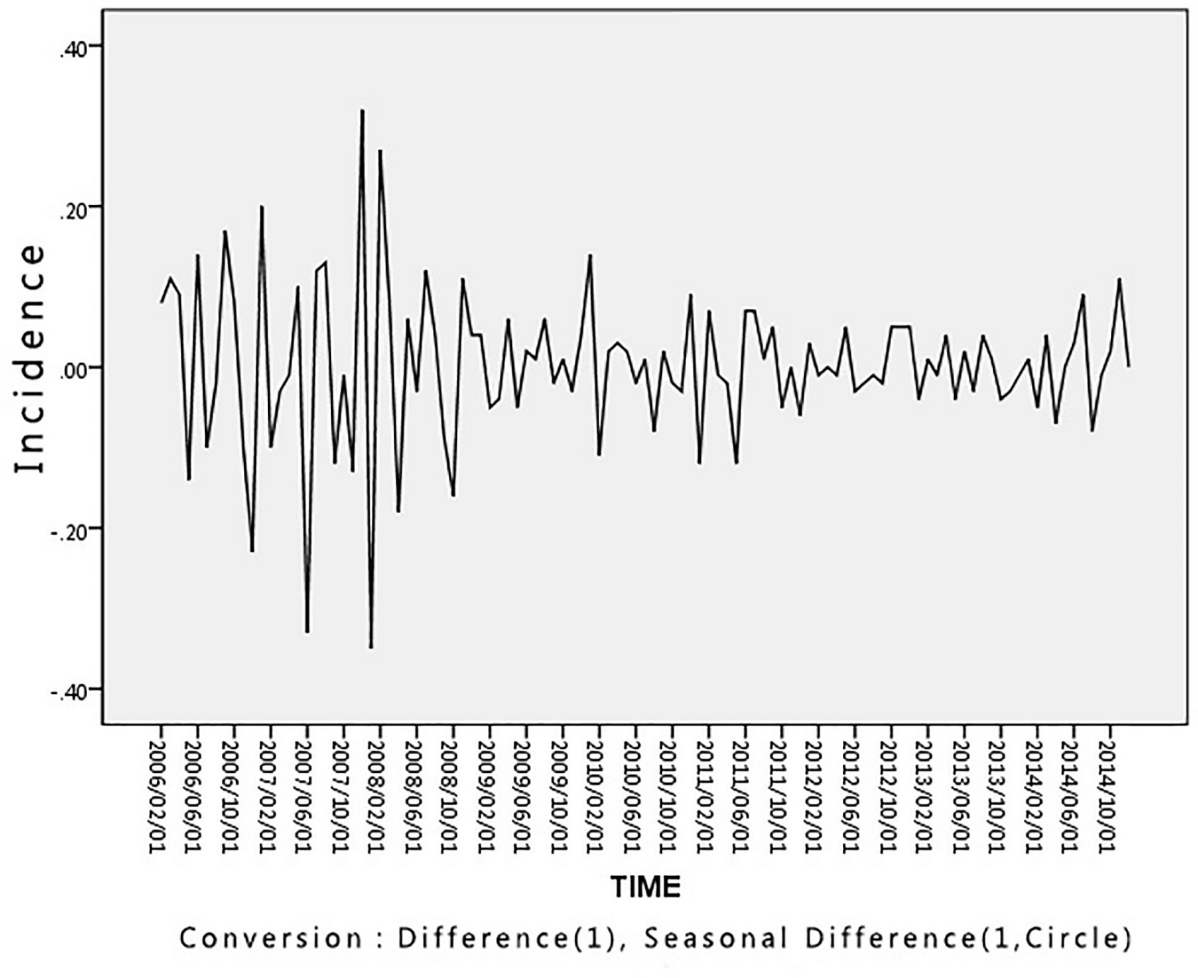
Time series results using first-order difference for the HFRS incidence data. The polyline is constructed using the collected HFRS incidence points after seasonal difference adjustment.

The results of the time series for the HFRS outbreaks data in Hubei Province from 2005 to 2014 using a first-order difference are shown in Fig 6

**Fig 7:**
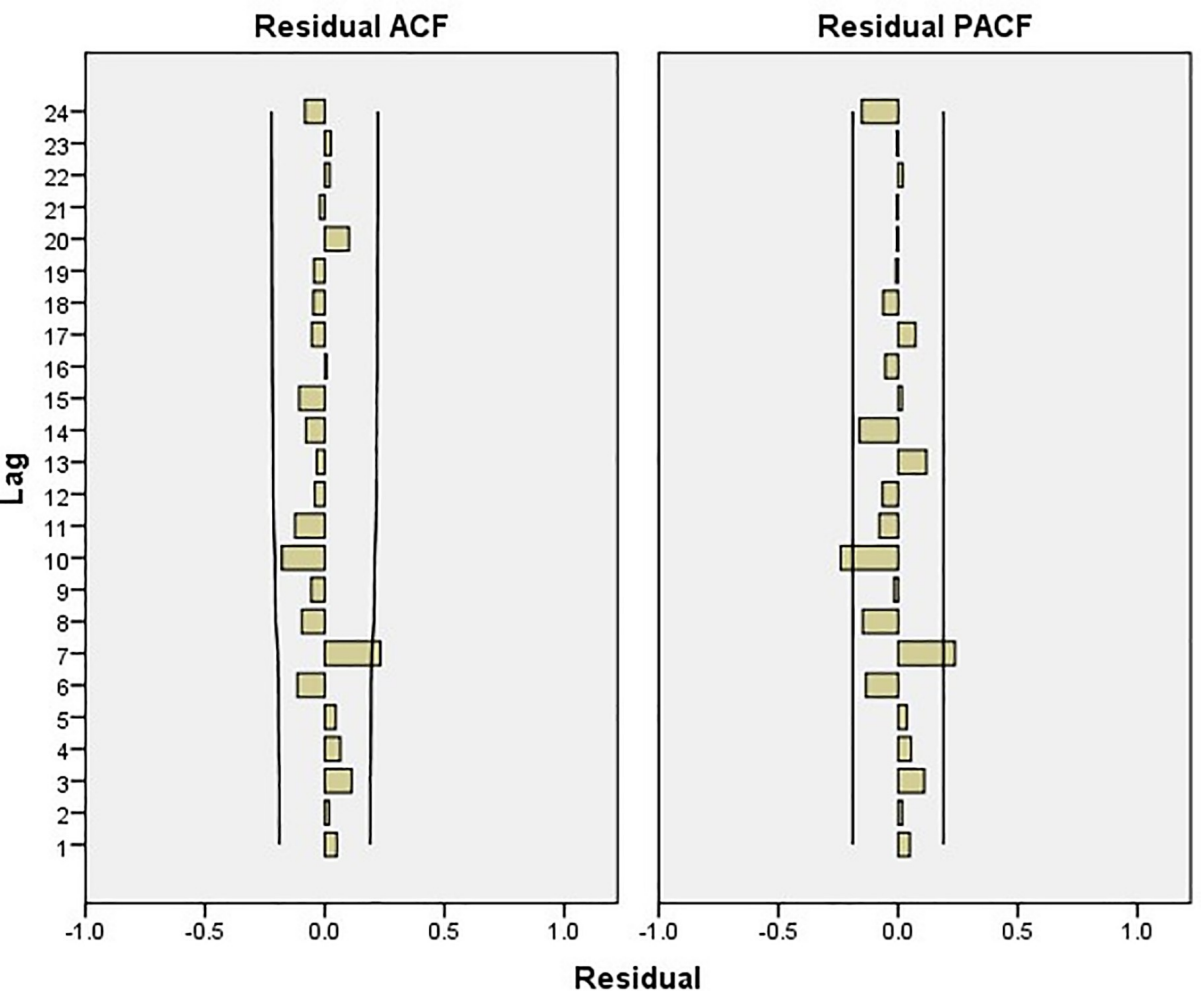
Stationarity analysis using first-order difference for the HFRS incidence data. The ACF and PACF values for HFRS incidence after seasonal difference adjustment for each lag.

Fig 7 presents the stationarity analysis results relating to HFRS incidence after using a first-order difference. The time series fluctuates around the value 0, indicating an overall uniform distribution. The ACF and PACF appear to be tailing off. It can be inferred from Fig 7. that, after taking the first-order difference into account, the time series shown in Fig 6. is a stationary time series. Therefore, for this paper we have chosen to use the first order difference to preprocess the data.

### Construction of the SD-STARIMA model and comparison with the ARIMA and STARIMA models

In this section we construct ARIMA, STARIMA and SD-STARIMA models using first-order difference for the time series relating to the HFRS incidence data.

#### ARIMA model

On the basis of first-order difference, the ARIMA(p,q) model can be defined as:

$$z(t)=\phi_{1}z(t-1)+\ldots +\phi_{p}z(t-p)+\varepsilon (t)+\theta_{1}\varepsilon (t-1)-\ldots \theta_{q}\varepsilon (t-q)$$ (6)

On the basis of the ACF and PACF across different time lag values, p = 4 and q = 2 were selected as the values for this model. The autoregressive coefficient, moving average coefficient and test parameters are shown in Table 3.

**Table 3:** ARIMA model parameters.

		Estimate	t	Sig.
Constant		-.028	-1.598	.003
AR	Lag 1	1.796	16.278	.000
	Lag 2	-1.037	-4.945	.000
	Lag 3	.232	1.114	.008
	Lag 4	-.084	-.773	.002
MA	Lag 1	1.706	23.993	.000
	Lag 2	-.940	-12.853	.000

#### STARIMA model

For the STARIMA model, a spatial weight matrix had to be established first of all. First-order spatial neighborhood matrices and second-order spatial domain matrices of 73*73 were obtained according to the spatial neighborhood relationship of 73 counties in Hubei Province (there are actually 76 counties, but 73 were used as samples and the other 3 for validation). The core diagonal elements of the first-order adjacency matrix are 0. There are no adjacent spatial units if the non- diagonal elements are 0. 1 indicates that there are adjacent spatial units. The first- and second-order spatial neighborhood matrix can be obtained on the basis of the specific adjacency unit according to the row and column identifying the elements and the line standardization.

The space-time autocorrelation coefficients and space-time partial autocorrelation coefficients are then calculated for the HFRS outbreaks data incidence series (before seasonal difference). The calculated results are shown in Tables 4 and 5.

**Table 4:** Autocorrelation function values of HFRS incidence before seasonal difference.

spatial lags(h) time lags(k)	0	1	2
1	0.015	-0.051	-0.060
2	0.118	-0.020	0.041
3	0.156	0.036	0.014
4	0.13	0.051	0.062
5	0.014	-0.122	-0.123
6	0.115	0.072	0.105
7	0.087	-0.048	-0.069
8	0.144	0.039	0.022
9	0.131	0.059	0.054
10	0.015	-0.084	-0.077
11	0.046	0.003	0.006
12	0.073	0.071	0.081

**Table 5:** Partial Autocorrelation function values of HFRS incidence before seasonal difference.

spatial lags (h) time lags (k)	0	1	2
1	0.115	-0.501	-0.660
2	0.106	-0.363	-0.521
3	0.135	-0.245	-0.458
4	0.094	-0.089	-0.257
5	-0.036	-0.185	-0.264
6	0.079	-0.132	-0.184
7	0.046	-0.182	-0.211
8	0.115	-0.138	-0.290
9	0.086	0.013	-0.171
10	-0.061	-0.042	-0.117
11	-0.008	-0.068	-0.162
12	0.021	0.009	-0.043

The ACF values are truncated after time lag 4 and for all of the spatial lags. The PACF values are truncated after time lag 3 and for all of the spatial lags. In that case, a STARIMA (4,3) model can be constructed using the results in Table 6.

**Table 6:** STARMA (4,3) model.

		Estimate	t	Sig.
Constant		.170	6.851	.000
AR	Lag 1	-1.243	-7.854	.000
	Lag 2	-.252	-1.348	.008
	Lag 3	.421	2.580	.001
	Lag 4	.107	.748	.006
MA	Lag 1	-2.197	-17.866	.000
	Lag 2	-1.989	-9.528	.000
	Lag 3	-.727	-6.060	.000

#### SD-STARIMA model

Tables 7 and 8 present the calculated values for the space-time autocorrelation coefficient and space-time partial autocorrelation coefficient after applying the first-order difference series to the HFRS outbreaks data.

**Table 7:** ACF values of HFRS incidence after seasonal difference adjustment.

spatial lags (h) time lags (k)	0	1	2
1	0.359	0.286	0.248
2	0.287	0.006	0.069
3	0.297	0.099	0.033
4	0.234	0.028	0.026
5	0.045	0.017	0.015
6	0.033	0.031	0.050
7	0.029	0.022	0.020
8	-0.029	-0.012	-0.002
9	-0.002	-0.028	-0.009
10	-0.003	-0.005	-0.030
11	0.017	-0.026	-0.034
12	-0.004	-0.019	-0.042

**Table 8:** PACF values of HFRS incidence after seasonal difference adjustment.

spatial lags (h) time lags (k)	0	1	2
1	0.359	0.359	0.248
2	0.132	0.052	0.021
3	0.045	0.045	0.041
4	0.040	0.030	0.016
5	0.010	0.020	-0.038
6	-0.007	-0.017	-0.035
7	0.010	0.070	0.003
8	-0.040	-0.040	-0.044
9	-0.013	-0.033	-0.016
10	-0.010	-0.030	-0.044
11	0.027	0.017	0.033
12	-0.005	-0.015	-0.009

Looking at the results in Tables 7 and 8, it can be seen that both the AFC and PACF are tailing off. This confirms that this is a STARIMA model. A candidate time autocorrelation average moving model as in STARIMA (1,1) can now be got using the transformation status of the AFC and PACF. The STARIMA (1,1) model can be expressed formally as:

$$z(t)=\varphi_{10}z(t-1)+\varphi_{11}W^{1}z(t-1)+\varphi_{12}W^{2}z(t-1)+\varepsilon (t)-\theta_{10}\varepsilon (t-1)-\theta_{11}W^{1}\varepsilon (t-1)-\theta_{12}W^{2}\varepsilon (t-1)$$ (7)

A maximum likelihood estimate is made for the STARIMA (1,1) model to obtain its parameter estimation values and hypothesis test values. The results are shown in Table 9.

**Table 9:** Parameter estimation and test results for the SD-STARMA model.

	*φ*_*10*_	*φ*_*11*_	*φ*_*12*_	*θ*_*10*_	*θ*_*11*_	*θ*_*12*_
Parameters	1.351	0.025	-0.125	0.852	0.113	-0.112
T-test	35.845	15.123	8.559	3.325	1.046	-0.015
P value	0.000	0.001	0.000	0.015	0.235	0.441

### HFRS incidence prediction

**Fig 8:**
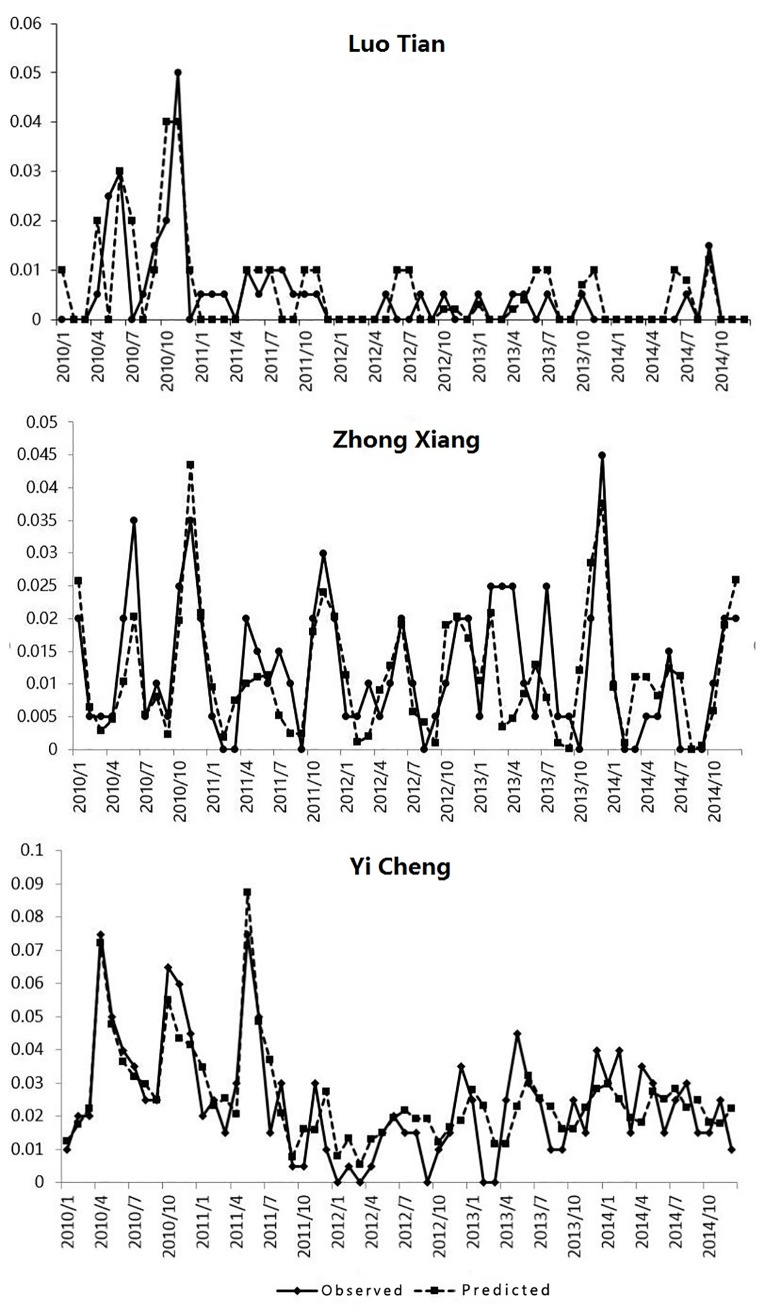
Comparison between observed and predicted values of HFRS incidence in Luotian, Zhongxiang and Yicheng counties from 2010–2014. The continuous lines with points represent the observed HFRS incidence and the dotted lines with points represent the predicted values for HFRS incidence for each specific date.

Highly representative areas or areas with a high incidence of the disease were used to validate the model. Luotian, Zhongxiang and Yicheng counties were used to undertake a comparison. The observed values and predicted values for these three counties are presented in Fig 8. It can be seen that the two values are very close, indicating that the prediction results are reliable.

We also evaluated the prediction results and general performance of the ARIMA, STARIMA and SD-STARIMA models to assess their relative effectiveness. Table 10 shows the correlation coefficient (R), Mean Absolute Percentage Error (MAPE), Root Mean Square Error (RMSE), Average Absolute Error (MAE) and Classic Akaike Information Criterion(AIC) for each of the models. It can be seen from the table that the SD-STARIMA model is more reliable and that the error between its predicted values and actual observed values is smaller. Overall, then, we can conclude as follows:

1. The data relating to HFRS incidence in Hubei Province has a fluctuating distribution curve and is quite different from other statistically sampled data in terms of its space and time distribution features, which are characterized by an obvious seasonal distribution. An SD-STARIMA model was therefore introduced that is able to adjust for seasonal difference and thus fit the data incorporating seasonal distribution trends more effectively.
2. The data for HFRS incidence in Hubei Province has both spatial and temporal characteristics. The SD-STARIMA model has both spatial and temporal features that are thus able to explain and simulate the HFRS tendencies in Hubei, with a spatial-temporal weight matrix being used to quantify the influence from the neighboring counties. We found that the SD-STARMA model has a higher degree of fit as a result of its implementation of time-space autocorrelation than would be the case with time autocorrelation alone.
3. Although the overall trend for HFRS incidence is consistent across every county in Hubei Province, the time series for the different counties is still different because of various impacting factors such as the local environment and human demography. The SD-STARIMA model is able to combine not only historical influences, but also the spatial and temporal impact from neighboring counties to evaluate the tendencies for HFRS incidence for any one specific county.

**Table 10:** Results for both fit and general prediction performance for the ARIMA, STARIMA and SD-STARIMA models.

Location	Model	R	MAPE	RMSE	MAE	Classic AIC
	SD-STARIMA	0.84	10.3	0.004	0.001	2548.12
Luotian	STARMA	0.75	18.6	0.009	0.002	2921.15
	ARIMA	0.48	57.3	0.094	0.068	3152.84
	SD-STARIMA	0.81	13.2	0.021	0.003	2651.72
Zhongxiang	STARMA	0.73	21.3	0.012	0.005	2712.50
	ARIMA	0.48	57.3	0.094	0.068	3524.21
	SD-STARIMA	0.73	9.12	0.001	0.012	2212.33
Yicheng	STARMA	0.71	22.4	0.018	0.027	2651.12
	ARIMA	0.48	57.3	0.094	0.068	3022.44

Having arrived at our results, we also compared them, to previous studies relating to HFRS analysis and prediction. Zhang at al., for instance, used a basic Poisson regression method to examine the potential impact of climate variability on the transmission of HFRS [20]. They incorporated climatic variables across a range of lags into a basic Poisson regression model that effectively eliminated the lagged effect of the climatic variables on the number of HFRS cases. However, spatial influences and spatial lag for the HFRS data were not considered, potentially overlooking a significant set of influencing factors.

Li et al. have used a GWR (geographically weighted regression) model to identify the impact of environmental factors and social-economic factors on the spatiotemporal heterogeneity of HFRS in China [42]. In this model, spatial characteristics are taken into account when undertaking the GWR-based analysis. However, this model suffers from the opposite flaw to the one above: temporal correlation is also a key influencing factor for HFRS cases in Hubei Province. Thus, by overlooking the temporal factors, this may similarly undermine the accuracy of the estimated results.

## Conclusion

Time series-based approaches have commonly been used in the past to predict the trends in HFRS epidemics, with ARIMA models standing as prime example. As a result of their capacity to capture both spatial and temporal variation, simulation results based on STARIMA models have been found to be more accurate than the results provided by non-spatial models like ARIMA. However, because there are also seasonal characteristics relating to the HFRS epidemics in Hubei Province, we developed a new model named SD-STARIMA that is able to incorporate adjustments for seasonal differences into space-time series analysis of HFRS outbreaks. We compared the estimates produced by ARIMA, STARIMA and SD-STARIMA for HFRS incidence data for Hubei Province and found that the SD-STARIMA model more closely predicted observed trends.

In conclusion, our examination of various possible models in this paper demonstrated the importance of analyzing seasonal differences in relation to HFRS epidemics because of the disease’s seasonal characteristics. On top of this, we found that first-order differences most closely reflect the stability data and bimodal distribution characteristics of the disease. We then constructed a first-order difference based SD-STARIMA model that is able to make accurate predictions using both space-time autocorrelation coefficients and space-time partial autocorrelation coefficients.

To validate the proposed approach, we used data relating to three counties that have a higher incidence of HFRS in Hubei Province (Luotian, Zhongxiang and Yicheng). According to the results, the SD-STARIMA model is more accurate than the ARIMA and STARIMA models and is generally much better for counties that are consistent with overall distribution trends. In that case, the SD-STARIMA model proposed in this paper has been proven to be more reliable for predicting HFRS epidemics in Hubei Province and has the potential to be more widely used for the prediction of epidemics.
